# HbA1c variability and risk of incident heart failure: A systematic review and meta-analysis

**DOI:** 10.17305/bb.2025.13376

**Published:** 2025-12-19

**Authors:** Guo Mujiafu Qiao Longbatu, La Xinamujila, Bao Burie

**Affiliations:** 1 Cardiology Department, International Mongolian Hospital of Inner Mongolia, Hohhot 010020, China; 2 College of Mongolian Medicine, Inner Mongolia Minzu University, Tongliao 028000, China; 3 NMPA Key Laboratory for Quality Control of Traditional Chinese Medicine, Inner Mongolia Minzu University, Tongliao 028000, China

**Keywords:** Glycated hemoglobin, variability, heart failure, risk factor, meta-analysis

## Abstract

Visit-to-visit variability in glycated hemoglobin (HbA1c) reflects long-term instability in glycemic control, potentially contributing to cardiovascular complications. However, the association between HbA1c variability and heart failure (HF) risk remains unclear. This meta-analysis aimed to quantify the relationship between HbA1c variability and the risk of incident HF in adults. A systematic search of PubMed, Embase, and Web of Science was conducted to identify relevant studies. Observational studies and post-hoc analyses of clinical trials evaluating the association between visit-to-visit HbA1c variability and incident HF were included. Random-effects models were employed to pool hazard ratios (HRs) with 95% confidence intervals (CIs), accounting for potential heterogeneity. A total of nine studies (*n* ═ 342,123) were included in the analysis. Overall, high HbA1c variability was associated with an increased risk of HF (pooled HR = 1.78, 95% CI: 1.39–2.27, *P* < 0.001; *I*^2^ ═ 87%). Sensitivity analyses restricted to patients with type 2 diabetes (T2D) (HR = 1.73, 95% CI: 1.35–2.22), high-quality studies (HR = 1.82, 95% CI: 1.32–2.50), or studies adjusting for mean HbA1c (HR = 1.68, 95% CI: 1.31–2.16) produced consistent results. Subgroup analyses indicated a stronger association in prospective cohorts (HR = 2.51) compared to retrospective or post-hoc studies (*P* for subgroup difference < 0.001). Meta-regression analysis revealed no significant modifying effects of age, sex, follow-up duration, or study quality (*P* all > 0.05). In conclusion, greater visit-to-visit HbA1c variability may be associated with an increased risk of incident HF, underscoring the prognostic importance of maintaining stable long-term glycemic control in patients with T2D.

## Introduction

Heart failure (HF) poses a significant global health challenge, affecting over 64 million individuals worldwide and serving as a leading cause of hospitalization and mortality among older adults [1, 2]. Despite advancements in pharmacologic and device-based therapies, the prognosis for HF remains poor, with five-year mortality rates approaching 50% [3]. Conventional risk factors such as hypertension, diabetes mellitus, obesity, and coronary artery disease only partially account for HF occurrences, with a considerable proportion of cases developing in individuals without overt cardiac disease [4]. Consequently, identifying novel and modifiable risk factors is crucial for enhancing early prevention and risk stratification. Among these factors, metabolic dysregulation, particularly abnormal glucose metabolism, has gained recognition as a significant contributor to cardiac remodeling, fibrosis, and dysfunction [5]. Chronic hyperglycemia is known to elevate the risk of HF through mechanisms involving oxidative stress, endothelial injury, and myocardial fibrosis [6]. Recent evidence also suggests that glycemic fluctuations may exert additional deleterious cardiovascular effects independent of sustained hyperglycemia, potentially through the repetitive activation of oxidative and inflammatory pathways that impair myocardial energetics and vascular integrity [7, 8].

Long-term glucose fluctuations can be objectively assessed using serial measurements of glycated hemoglobin (HbA1c), which reflects average glycemia over the preceding two to three months [9]. The variability of HbA1c across clinical visits—termed visit-to-visit HbA1c variability—has emerged as a reliable index of long-term glycemic instability [10]. Several statistical measures are utilized to quantify HbA1c variability, including standard deviation (SD), coefficient of variation (CV), average real variability (ARV), variability independent of the mean (VIM), and adjacent SD (ASV), calculated from at least three separate HbA1c measurements during follow-up [11, 12]. Unlike mean HbA1c, which reflects average exposure to hyperglycemia, these indices capture dynamic fluctuations that may better represent the biological stress imposed on the cardiovascular system [13]. Although recent studies indicate that greater HbA1c variability is associated with an increased risk of adverse cardiovascular events—including myocardial infarction, stroke, and all-cause mortality—findings regarding its relationship with incident HF remain inconsistent [14, 15]. Therefore, this meta-analysis aims to quantitatively evaluate the association between visit-to-visit HbA1c variability and the risk of incident HF in adults, clarifying the strength and consistency of this relationship and providing further insights into the prognostic significance of long-term glycemic instability.

## Materials and methods

This meta-analysis adhered to the Preferred Reporting Items for Systematic Reviews and Meta-Analyses (PRISMA) 2020 guidelines [16] and the Cochrane Handbook for Systematic Reviews and Meta-Analyses [17] for protocol design, data extraction, statistical analysis, and results reporting. The study protocol was registered in International Prospective Register of Systematic Reviews (PROSPERO) under ID CRD420251167937, with no methodological deviations occurring during the review process.

### Literature search

Relevant studies for this meta-analysis were identified through a comprehensive search in PubMed, Embase, and Web of Science, employing a broad range of search terms: (1) “glycosylated hemoglobin” OR “HbA1c”; (2) “variability” OR “variation” OR “fluctuation” OR “coefficient of variation” OR “standard deviation”; (3) “heart failure” OR “cardiac failure” OR “cardiac dysfunction”; and (4) “incidence” OR “risk” OR “cohort” OR “longitudinal” OR “prospective” OR “retrospective” OR “prospectively” OR “retrospectively” OR “followed” OR “follow-up.” The search was restricted to human studies and full-length articles published in English in peer-reviewed journals. Additionally, references from relevant original and review articles were manually screened for further eligible studies. The search spanned from database inception to August 30, 2025. The full search strategy for each database is detailed in Supplemental File 1. Grey literature sources were excluded due to concerns about data reliability in observational meta-analyses. Trial registries were not searched as our review focused on published cohort studies and post-hoc analyses rather than randomized trials.

### Inclusion and exclusion criteria

Eligibility criteria for studies were established based on the PICOS framework:

P (patients): General adult population (≥18 years) without HF at baseline, including both diabetic and non-diabetic individuals.

I (exposure): Participants exhibiting high visit-to-visit HbA1c variability at baseline, with parameters and cutoff values for defining high HbA1c variability consistent with those used in the original studies.

C (comparison): Participants with low visit-to-visit HbA1c variability at baseline.

O (outcome): Incidence of HF during follow-up, compared between participants with high vs low HbA1c variability at baseline, with diagnostic methods consistent with those in the original studies.

S (study design): Observational studies with longitudinal follow-up, such as cohort studies, nested case-control studies, or post-hoc analyses of clinical trials.

Studies were excluded if they were reviews, editorials, meta-analyses, preclinical research, involved pediatric patients, did not assess visit-to-visit HbA1c variability, or did not report the incidence of HF. Studies focusing on short-term glycemic fluctuations, such as daily glucose variability that did not utilize HbA1c or metrics from continuous glucose monitoring, were also excluded. In cases of population overlap, the study with the largest sample size was selected for inclusion in the meta-analysis.

### Study quality assessment and data extraction

Two authors independently conducted the literature search, study selection, quality assessment, and data extraction, resolving discrepancies through discussion with the corresponding author. Formal inter-rater agreement statistics were not recorded. Study quality was evaluated using the Newcastle–Ottawa Scale (NOS) [18], which assesses selection, confounding control, and outcome measurement, with scores ranging from 1 to 9, where 9 indicates the highest quality. Studies with NOS scores of 8 or above are deemed high quality. Data extracted for analysis included study characteristics (author, year, country, and study design), participant characteristics (source of the population, number of participants, age, sex, and diabetic status), exposure characteristics (parameters for evaluating HbA1c variability, cutoffs, and times of HbA1c measurements), follow-up durations, outcome characteristics (methods for validating HF outcomes and number of patients with newly developed HF during follow-up), and variables adjusted in estimating the relationship between HbA1c variability and HF risk. For each included study, we extracted only the adjusted hazard ratio (HR) specific to incident HF, as defined in the original article or its supplemental materials. HF endpoints included adjudicated HF events, HF hospitalization, or International Classification of Diseases (ICD)-based incident HF, depending on each study’s protocol. Composite cardiovascular or mortality outcomes were not included. All outcome definitions, adjudication statuses, and extraction locations (table/figure) were independently cross-checked by two reviewers.

### Statistical analyses

The relationship between HbA1c variability and HF in adults was reported as HRs with corresponding 95% confidence intervals (CIs). This analysis compared participants with high vs low HbA1c variability at baseline. When studies provided multiple validated metrics of HbA1c variability, we extracted a single effect estimate per study to ensure statistical independence, in accordance with Cochrane recommendations [17]. Given the absence of a universally accepted primary variability metric and the complementary nature of different indices (e.g., SD, CV, ARV, VIM, ASV, and HbA1c variability score [HVS]), we refrained from designating a single preferred metric *a priori*. Instead, we included one representative adjusted estimate per study and conducted predefined subgroup analyses by variability metric to assess consistency across measures. If a study reported multiple metrics for HbA1c variability, we adhered to a predefined rule: the adjusted HR associated with the largest reported effect size was selected. This rule was established prior to data synthesis and consistently applied across all studies. HRs and their standard errors were derived from the 95% CIs or *P* values and were log-transformed to stabilize variance and normalize distribution [17]. To evaluate heterogeneity, we employed the Cochrane *Q* test and *I*^2^ statistics [19], interpreting *I*^2^ values of <25%, 25%–75%, and >75% as indicative of mild, moderate, and substantial heterogeneity among the included studies, respectively. The primary analysis utilized the DerSimonian–Laird estimator, with between-study heterogeneity quantified using the *I*^2^ statistic and between-study variance (τ^2^) [17]. To enhance robustness, we also conducted sensitivity analyses employing restricted maximum likelihood (REML) random-effects models with Hartung–Knapp adjustments. Prior to pooling, HRs were log-transformed [17]. For each primary meta-analysis, a 95% prediction interval (PI) was calculated to represent the expected range of effects in future comparable studies [17]. A random-effects model was utilized to synthesize results while accounting for variability across studies [17]. Sensitivity analyses were performed by sequentially excluding individual studies to assess the robustness of the findings [20]. Additionally, sensitivity analyses were limited to diabetic patients, high-quality studies (NOS ≥ 8), and studies that adjusted for mean HbA1c levels. Furthermore, predefined subgroup analyses were conducted to evaluate the impact of study characteristics on results, including study design (prospective vs retrospective or post-hoc analyses), geographic location (Asian vs Western countries), various parameters for HbA1c variability, and mean follow-up durations. Subgroups were defined using median values of continuous variables as cutoff points. Univariate meta-regression analyses were also performed to investigate whether continuous study characteristics, such as mean age of the population, proportion of men, mean follow-up durations, and NOS, influenced the association between HbA1c variability and HF risk [17]. All subgroup analyses and meta-regression models were prespecified but exploratory, conducted as univariate analyses only, without multiplicity correction; thus, these results should be interpreted with caution. Publication bias was assessed through funnel plots, visual asymmetry inspection, and Egger’s regression test [21]. Small-study effects were evaluated using Egger’s regression test and Duval and Tweedie’s trim-and-fill method [22]. A *P* value < 0.05 was considered statistically significant. Statistical analyses were conducted using RevMan (Version 5.3; Cochrane Collaboration, Oxford, UK) and Stata software (version 17.0; Stata Corporation, College Station, TX, USA).

**Figure 1.**
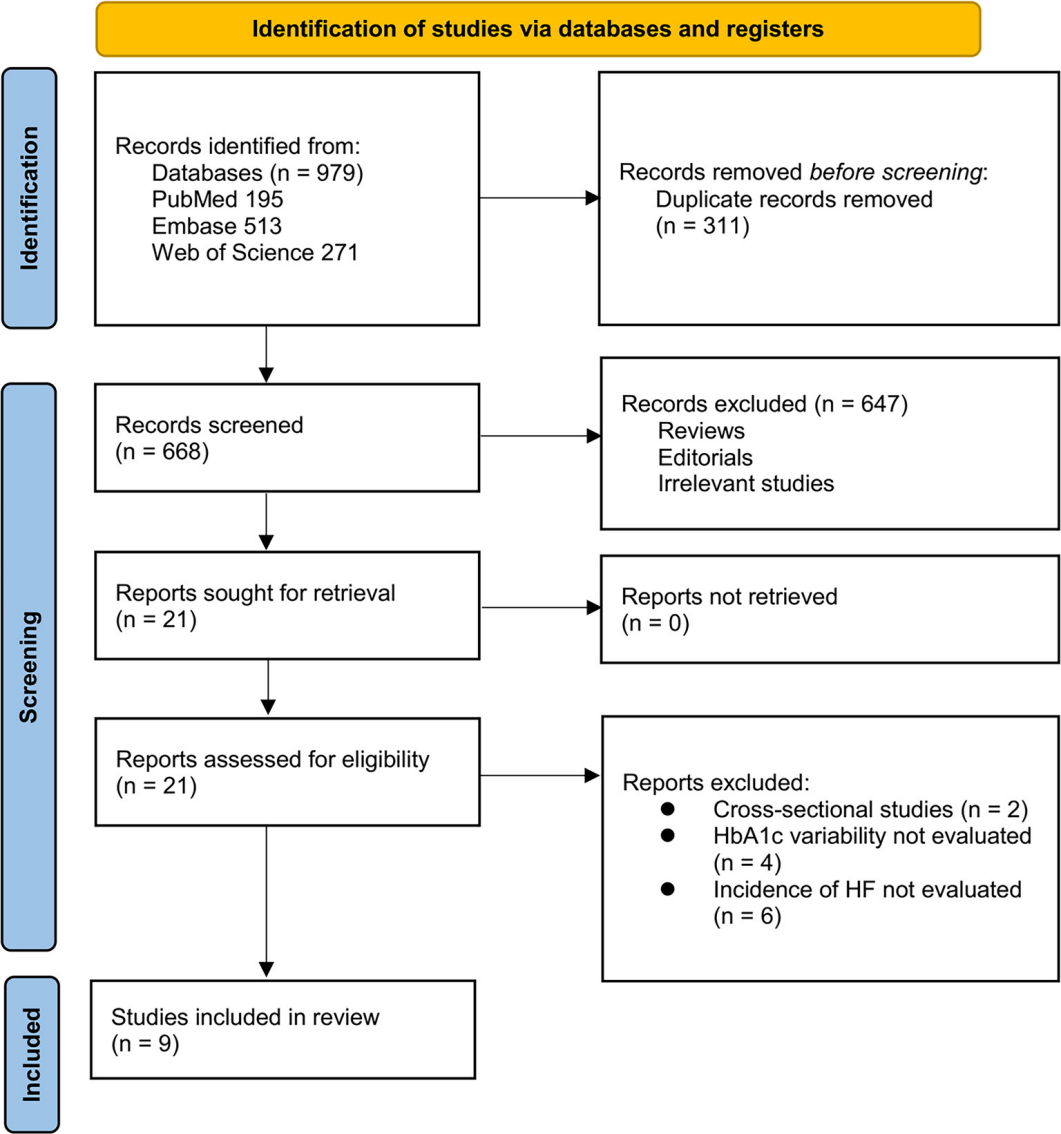
Flowchart of database search and study inclusion.

## Results

### Study identification

Figure 1 illustrates the study selection process. A total of 979 records were identified across three databases, with 311 duplicates removed. Following title and abstract screening, 647 articles were excluded for not meeting the meta-analysis criteria. The full texts of the remaining 21 studies were independently reviewed by two authors, resulting in the exclusion of 12 studies for reasons detailed in Figure 1. Ultimately, nine studies were included in the quantitative analysis [23–31].

### Overview of the study characteristics

Table 1 presents the main characteristics of the nine studies included in this meta-analysis, comprising two prospective cohort studies [27, 30], five retrospective cohort studies [23, 25, 28, 29, 31], and two post-hoc analyses of clinical trials [24, 26]. These studies were published between 2018 and 2025 and conducted across various geographic regions, including mainland China, Hong Kong (China), Taiwan (China), Thailand, Sweden, the United Kingdom, and the United States. Eight studies enrolled adult patients with type 2 diabetes (T2D) [23–29, 31], while one study included prediabetic or T2D patients with additional cardiovascular risk factors such as hypertension, obesity, or pre-existing atherosclerotic cardiovascular disease [30]. In total, 342,123 patients were included, with mean ages ranging from 58.4 to 65.3 years and the proportion of men varying from 37.9% to 61.9%. HbA1c variability was assessed using various indices, including SD, CV, VIM, ASV, ARV, and HVS. The exposure contrast between high and low variability was commonly defined by quartiles [24, 29–31] or quintiles [26], though some studies employed medians [23] or specific categorical cut-offs [25, 27]. The number of HbA1c measurements utilized to compute variability ranged from a minimum of 3 to an average of 12.7 per participant, with mean follow-up durations spanning 4.4–11.7 years. HF outcomes were identified through adjudicated clinical review processes [24, 26], validated diagnostic criteria from international guidelines [23], or HF-related hospitalizations recorded in institutional or national administrative databases [25, 27–31]. Across studies, the number of participants developing HF during follow-up ranged from 18 to 7,908. All studies performed multivariable-adjusted analyses, accounting for key confounders such as age, sex, body mass index, blood pressure, lipid profiles, kidney function, comorbidities, medication use, and mean HbA1c levels, thereby minimizing residual confounding.

### Study quality evaluation

As detailed in Table 2, the methodological quality of the included studies was evaluated using the NOS. NOS scores ranged from 7 to 9, indicating overall high methodological quality. Most studies achieved full scores for cohort representativeness, exposure ascertainment, exclusion of baseline HF, and adjustment for major confounders [24–31]. Two post-hoc analyses and one retrospective cohort study [24, 26, 31] attained the maximum NOS score of 9, reflecting robust design, standardized laboratory procedures, and adjudicated outcome assessment. Two large population-based registry studies [25, 27] and one single-center tertiary hospital cohort with claims-based outcome ascertainment [28] received a score of 8, with minor limitations related to the reliance on registry-coded rather than clinically confirmed HF diagnoses. One retrospective study also scored 8 due to potential bias in the representativeness of the exposed cohort [23]. Two additional studies were assigned a score of 7 because HF was not diagnosed through clinical evaluation and the follow-up durations were relatively short (<5 years) [29, 30]. Importantly, all studies demonstrated adequate follow-up and low attrition risk, supporting the reliability of pooled estimates linking long-term HbA1c variability to HF risk.

**Table 1:** Characteristics of the included studies

**Study**	**Country**	**Study design**	**Source of population**	**No. of participants**	**Mean age (years)**	**Men (%)**	**Parameters for HbA1c variability**	**Cutoffs of HbA1c variability**	**Times of HbA1c measured for variability**	**Mean follow-up duration (years)**	**Methods for validating HF outcome**	**No. of patients with HF**	**Variables adjusted**
Gu, 2018	China	RC	Patients with T2D and hypertension from a single institution	201	65.3	59.2	HbA1c-SD* and HbA1c-CV	Medians	Mean: 11.7	7.3	AHA/ACC diagnostic criteria for new-onset symptomatic HFpEF	18	Age, sex, SBP, DBP, HbA1c-mean, eGFR, BMI, duration of T2D and hypertension, AF, medical treatment (Calcium blocker, ACEI/ARB, Beta-blockers, Statin, Sulfonylurea, Metformin, α-GI, Thiazolidinedione, Insulin), LAD, LVMI, E/E’, and LVEF
Kaze, 2020	USA	Post-hoc analysis of an RCT	Overweight or obese adults with T2D	3560	58.4	37.9	HbA1c-SD*, HbA1c-CV, HbA1c-VIM, and HbA1c-ASV	Q4:Q1	Mean: 4	6.8	Adjudicated incident HF events, as per the predefined process in the Look AHEAD trial	91	Age, sex, race/ethnicity, randomization arm, BMI, current smoking, alcohol drinking, use of antihypertensive medications, average ratio of total to HDL-c, eGFR, duration of diabetes, average SBP, and average HbA1c
Segar, 2020	USA	Post-hoc analysis of an RCT	Adults withT2D and high cardiovascular risk or established CVD	8576	62.4	61.9	HbA1c-SD, HbA1c-CV, and HbA1c-ASV*	Q5:Q1	Median: 8	6.4	Adjudicated incident HF hospitalization or death due to HF by an independent committee	388	Age, sex, race, education, intensive glycemic control treatment group, history of CVD, traditional cardiovascular risk factors (systolic BP, BMI, cigarette use, alcohol use, total cholesterol, serum creatinine, LDL-c, HDL-c), medication use (ARB, ACE inhibitor, β-blocker, loop diuretic, thiazide diuretic, calcium channel blocker, insulin, sulfonylurea, biguanide, meglitinide, α-glucosidase inhibitor), and mean HbA1c
Li, 2020	UK	RC	Adults with newly diagnosed T2D	19,059	63.3	54.6	HVS	HVS 80–100 vs. 0–20	Median: 12	6.8	Hospitalization or death from HF (as per electronic health records)	853	Age, sex, calendar year, Scottish Index of Multiple Deprivation, smoking, hypertension, BMI, HDL-c, eGFR, antiplatelet therapy, and CCI
Wan, 2020	Hong Kong (China)	PC	Hong Kong Hospital Authority (HA) electronic health records; primary care patients with T2D	147,811	64.2	46.0	HbA1c-SD	≥3.0% vs. 0%–0.24%	Mean: 3.2	7.4	Hospital Authority electronic health records and death registry, using ICPC-2 and ICD-9/10 codes	7908	Age, sex, smoking status, duration of diabetes, BMI, systolic and diastolic BP, LDL-c, eGFR, use of metformin, sulphonylureas, other oral diabetic drugs, insulin, anti-hypertensive drugs, lipid-lowering agents, CCI, and mean HbA1c
Lin, 2021	Taiwan (China)	RC	T2D patients from Diabetes Shared Care Program at a tertiary hospital (China Medical University Hospital)	3824	58.5	50.2	HbA1c-SD	T3:T1	At least 3 measurements within a 12–24 month baseline period	11.7	HF by National Health Insurance claim database using ICD-9-CM and ICD-10-CM codes	315	Age, sex, diabetes duration, BMI, systolic BP, total cholesterol, triglyceride, HDL-c, LDL-c, eGFR, CAD, hypertension, stroke, and use of sulfonylureas, metformin, thiazolidinediones, insulin, statin, antiplatelet agents, warfarin, ACEIs, ARB, beta-blockers, CCB, diuretics, alpha-blockers, and mean HbA1c
Ceriello, 2022	Sweden	RC	T2D patients from Swedish National Diabetes Register	101,533	64.3	55.6	HbA1c-SD	Q4:Q1	At least 5 measurements during a 3-year exposure phase	4.4	National registry data using ICD-9 and ICD-10 codes (Hospitalization for HF)	NR	Age, sex, duration of diabetes, body weight, smoking, mean HbA1c, systolic and diastolic BP, total cholesterol, HDL-c, LDL-c, triglycerides, albuminuria, eGFR, retinopathy, treatment for diabetes, hypertension, dyslipidemia, and aspirin
Manosroi, 2023	Thailand	PC	Thai patients aged >45 years with high atherosclerotic risk (Prediabetes or T2D)	3811	64.7	46.6	HbA1c-SD	Q4:Q1	Median: 3	4.5	Hospitalization for HF (as part of the 4P-MACE outcome)	109	Age, sex, educational level, BMI, established ASCVD status, systolic BP, smoking status, mean HbA1c, lipid profiles, creatinine level, number of HbA1c measurements, antihypertensive medications, diabetes medications, lipid-lowering agents, and antiplatelet/anticoagulants
Hsiao, 2025	Taiwan (China)	RC	T2D patients from The Chang Gung Research Database	53,748	63.7	50.7	HbA1c-ARV	Q4:Q1	Mean: 12.7	6.2	HF hospitalization, defined as a principal discharge diagnosis of HF plus at least one treatment during hospitalization (diuretics, nitrites, or inotropic agents)	1995	Age, sex, BMI, smoking, all comorbidities, baseline renal function, all medications, average lipid profiles, average vital signs (systolic/diastolic BP, heart rate), hypoglycemia, hyperglycemia, and the average HbA1c level

*, parameter used in the main meta-analysis. Abbreviations: RC: Retrospective cohort; PC: Prospective cohort; RCT: Randomized controlled trial; T2D: Type 2 diabetes; HbA1c: Glycated hemoglobin; SD: Standard deviation; CV: Coefficient of variation; VIM: Variability independent of the mean; ASV: Adjacent standard deviation; HVS: HbA1c variability score; HF: Heart failure; HFpEF: Heart failure with preserved ejection fraction; AHA/ACC: American Heart Association/American College of Cardiology; eGFR: Estimated glomerular filtration rate; BMI: Body mass index; SBP: Systolic blood pressure; DBP: Diastolic blood pressure; AF: Atrial fibrillation; ACEI: Angiotensin-converting enzyme inhibitor; ARB: Angiotensin II receptor blocker; LAD: Left atrial diameter; LVMI: Left ventricular mass index; LVEF: Left ventricular ejection fraction; HDL-c: High-density lipoprotein cholesterol; LDL-c: Low-density lipoprotein cholesterol; CAD: Coronary artery disease; CVD: Cardiovascular disease; ASCVD: Atherosclerotic cardiovascular disease; CCI: Charlson comorbidity index; CCB: Calcium channel blocker; ICD: International Classification of Diseases; ICPC: International Classification of Primary Care; NR: Not reported.

**Table 2:** Evaluation of study quality using the Newcastle–Ottawa Scale: Justifications

**Study**	**Representativeness of the exposed cohort**	**Selection of the non-exposed cohort**	**Ascertainment of exposure**	**Outcome not present at baseline**	**Control for age and sex**	**Control for other confounding factors**	**Assessment of outcome**	**Long enough follow-up duration**	**Adequacy of follow-up of cohorts**	**Total**
Gu, 2018	0 point. The exposed cohort was drawn from a hospital medical record database. Unknown if the patients were consecutively or randomly enrolled.	1 point. The non-exposed cohort (low HbA1c variability group) was drawn from the same source as the exposed cohort (high HbA1c variability group)	1 point. HbA1c was measured using a standardized, DCCT-aligned laboratory method (high-performance liquid chromatography)	1 point. Patients with symptomatic heart failure at baseline were explicitly excluded.	1 point. Both age and sex were included in the initial univariate analysis and considered for the multivariable model	1 point. The study adjusted for numerous important confounders, including blood pressure, mean HbA1c, renal function, BMI, comorbidities, and medications	1 point. The outcome (symptomatic HFpEF) was assessed using strict, predefined, and accepted (AHA/ACC) diagnostic criteria involving both clinical and echocardiographic evidence	1 point. The median follow-up was 7.3 years, which is >5 years and sufficient for the outcome to occur	1 point. The study retrospectively enrolled patients who had been followed for at least 2 years, and follow-up information was obtained from a comprehensive medical record database, suggesting a low loss to follow-up	8
Kaze, 2020	1 point. The cohort was derived from a multi-center, randomized controlled trial (Look AHEAD) with a well-defined, prospective recruitment strategy	1 point. The non-exposed cohort (low HbA1c variability groups, e.g., Q1) was drawn from the same source as the exposed cohort (the trial population)	1 point. HbA1c was measured in a central laboratory using a standardized, high-performance method (ion-exchange HPLC), ensuring reliable exposure assessment	1 point. Participants with prevalent HF at baseline or during the first 36 months (the exposure assessment period) were explicitly excluded	1 point. Both age and sex were included in the multivariable regression models	1 point. The model adjusted for numerous important confounders, including race, BMI, smoking, blood pressure, lipids, renal function, diabetes duration, and crucially, the mean HbA1c level	1 point. The outcome (incident HF) was ascertained via a standardized and adjudicated process within the clinical trial, which is a high-quality method	1 point. The median follow-up was 6.8 years, which is >5 years and sufficient for the outcome to occur	1 point. As a post-hoc analysis of an RCT with a dedicated follow-up structure, the loss to follow-up is expected to be minimal	9
Segar, 2020	1 point. The cohort was derived from a large, multicenter randomized controlled trial (ACCORD) with a well-defined, prospective recruitment strategy	1 point. The non-exposed cohort (e.g., lower variability groups) was drawn from the same source as the exposed cohort (the trial population)	1 point. HbA1c was measured at a central laboratory using a standardized, NGSP-certified method at regular intervals, ensuring reliable exposure assessment	1 point. Participants with a history of HF or an HF event within the first 3 years of enrollment were explicitly excluded	1 point. Both age and sex were included in the multivariable regression models	1 point. The model adjusted for a comprehensive set of confounders, including cardiovascular history, risk factors, medications, baseline HbA1c, and crucially, the mean change in HbA1c and other time-updated cardiometabolic parameters	1 point. The outcome (incident HF) was adjudicated by an independent, blinded clinical events committee using predefined criteria, which is a high-quality method	1 point. The median follow-up for the outcome was 6.4 years, which is >5 years and sufficient	1 point. As a post-hoc analysis of an RCT with a dedicated follow-up structure, the loss to follow-up is expected to be minimal	9
Li, 2020	1 point. Population-based from SCI-DC database, includes all eligible newly diagnosed T2D patients	1 point. The non-exposed cohort was drawn from the same source as the exposed cohort	1 point. HbA1c measurements were extracted from electronic health records	1 point. Excluded patients with HF within first 3 years of diagnosis	1 point. Both age and sex were included in the multivariable regression models	1 point. Adjusted for multiple confounders (smoking, BMI, eGFR, deprivation, etc.)	0 point. HF defined by hospitalization or death, from validated records, not by clinically diagnosed HF	1 point. The median follow-up for the outcome was 6.8 years, which is >5 years and sufficient	1 point. Low attrition due to use of national registry data	8
Wan, 2020	1 point. Population-based, using the Hong Kong HA database which covers >90% of local patients with chronic diseases	1 point. The non-exposed cohort was drawn from the same source as the exposed cohort	1 point. HbA1c measured from standardized laboratory tests within the HA system	1 point. Patients with a prior diagnosis of CVD at baseline were explicitly excluded	1 point. Both age and sex were included in the multivariable regression models	1 point. Comprehensively adjusted for numerous clinical and treatment-related confounders, including mean HbA1c	0 point. Outcomes determined via linkage with robust electronic health records and the official death registry, not by clinically diagnosed HF	1 point. The median follow-up for the outcome was 7.4 years, which is >5 years and sufficient	1 point. Low risk of attrition due to the use of a comprehensive, population-wide administrative database	8
Lin, 2021	1 point. Consecutively enrolled from the hospital’s Diabetes Shared Care Program, representing a real-world clinical cohort	1 point. The non-exposed cohort was drawn from the same source as the exposed cohort	1 point. HbA1c measured extracted from electronic health records	1 point. Patients with a history of HF were excluded, and those who developed HF within 1 year of enrollment were also excluded to mitigate reverse causality	1 point. Both age and sex were included in the multivariable regression models	1 point. Comprehensively adjusted for a wide array of clinical, laboratory, and medication-related confounders	0 point. Outcome determined via linkage with a national claims database, not by clinically diagnosed HF	1 point. The median follow-up for the outcome was 11.7 years, which is >5 years and sufficient	1 point. Used a national database, suggesting minimal loss to follow-up	8
Ceriello, 2022	1 point. Population-based, using the Swedish National Diabetes Register which includes ∼90% of all patients with diabetes in Sweden	1 point. The non-exposed cohort was drawn from the same source as the exposed cohort	1 point. HbA1c measured by standard procedures as part of a national registry	1 point. Patients with prevalent macrovascular diseases (including HF) at baseline or during the exposure phase were excluded	1 point. Both age and sex were included in the multivariable regression models	1 point. Comprehensively adjusted for a very wide range of clinical, laboratory, and treatment-related confounders, including mean HbA1c	0 point. Outcomes determined via linkage with robust national registry data using standardized ICD codes, not by clinically diagnosed HF	0 point. The median follow-up for the outcome was 4.4 years, which is <5 years	1 point. Low risk of attrition due to the use of a comprehensive, national registry	7
Manosroi, 2023	1 point. The cohort is a multicenter, national registry (CORE-Thailand) designed to enroll patients with high atherosclerotic risk	1 point. The non-exposed cohort was drawn from the same source as the exposed cohort	1 point. Exposure (HbA1c variability) was ascertained from objective laboratory measurements (HbA1c SD) from patient records	1 point. The study is a longitudinal analysis of incident events. Patients were followed until they developed the outcome (HF hospitalization), died, or were censored	1 point. Both age and sex were included in the multivariable regression models	1 point. The model adjusted for numerous important confounders, including ASCVD status, BMI, smoking, mean HbA1c, renal function, lipid levels, and medication use	0 point. While not explicitly detailed, hospitalization for HF recorded, not by clinically diagnosed HF	0 point. The median follow-up for the outcome was 4.5 years, which is <5 years	1 point. The description states patients were followed until death, lost to follow-up, or censoring, suggesting a reasonable follow-up rate	7
Hsiao, 2025	1 point. The cohort is a large, multicenter, nationwide database (Chang Gung Research Database) that systematically collects data from all treated patients with T2D in that system	1 point. The “non-exposed” cohort (lowest quartile of HbA1c variability) was drawn from the same source population as the exposed cohort	1 point. Exposure (HbA1c variability) was ascertained from objective, serial laboratory measurements (HbA1c ARV) recorded in the medical database	1 point. The study explicitly excluded patients with a history of HF, myocardial infarction, or coronary intervention at baseline, ensuring the outcome was incident	1 point. Both age and sex were included in the multivariable regression models	1 point. The model adjusted for a very extensive set of confounders, including comorbidities, renal function, medications, lipid profiles, vital signs, hypoglycemia/hyperglycemia events, and crucially, the mean HbA1c level	1 point. HF was defined by a primary hospital discharge diagnosis plus the requirement for specific HF treatments, with the information of LVEF	1 point. The median follow-up for the outcome was 6.2 years, which is >5 years	1 point. The study used a comprehensive hospital database with a defined end-of-study date (Dec 31, 2018), suggesting minimal loss to follow-up	9

Abbreviations: HbA1c: Glycated hemoglobin; SD: Standard deviation; ARV: Average real variability; T2D: Type 2 diabetes; HF: Heart failure; HFpEF: Heart failure with preserved ejection fraction; AHA: American Heart Association; ACC: American College of Cardiology; HPLC: High-performance liquid chromatography; DCCT: Diabetes Control and Complications Trial; RCT: Randomized controlled trial; Look AHEAD: Action for Health in Diabetes trial; ACCORD: Action to Control Cardiovascular Risk in Diabetes trial; BMI: Body mass index; eGFR: Estimated glomerular filtration rate; CVD: Cardiovascular disease; ASCVD: Atherosclerotic cardiovascular disease; ICD: International Classification of Diseases; SCI-DC: Scottish Care Information–Diabetes Collaboration; HA: Hospital Authority; BP: Blood pressure; CAD: Coronary artery disease; HDL-c: High-density lipoprotein cholesterol; LDL-c: Low-density lipoprotein cholesterol; LVEF: Left ventricular ejection fraction.

### Meta-analysis results

**Figure 2.**
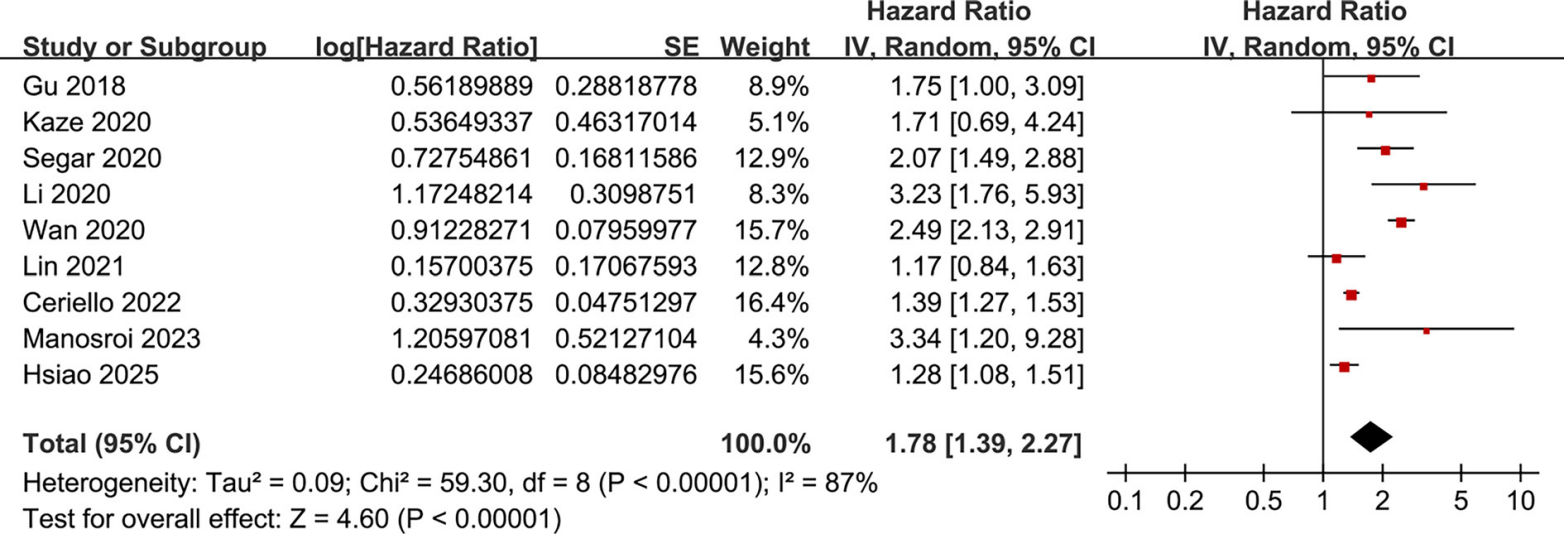
**Forest plot illustrating the association between high and low visit-to-visit HbA1c variability and the incidence of HF.** The adjusted HRs with 95% CIs from nine longitudinal studies were combined using an inverse-variance random-effects model. Squares denote individual study estimates, with their sizes proportional to study weight, while horizontal lines represent the 95% CIs. The diamond indicates the pooled effect (HR 1.78, 95% CI 1.39–2.27). The vertical line at HR = 1 signifies no association, and there was substantial between-study heterogeneity (*I*^2^ ═ 87%; τ^2^ ═ 0.09). Abbreviations: HbA1c: Glycated hemoglobin; HF: Heart failure; HR: Hazard ratio; CI: Confidence interval.

**Figure 3.**
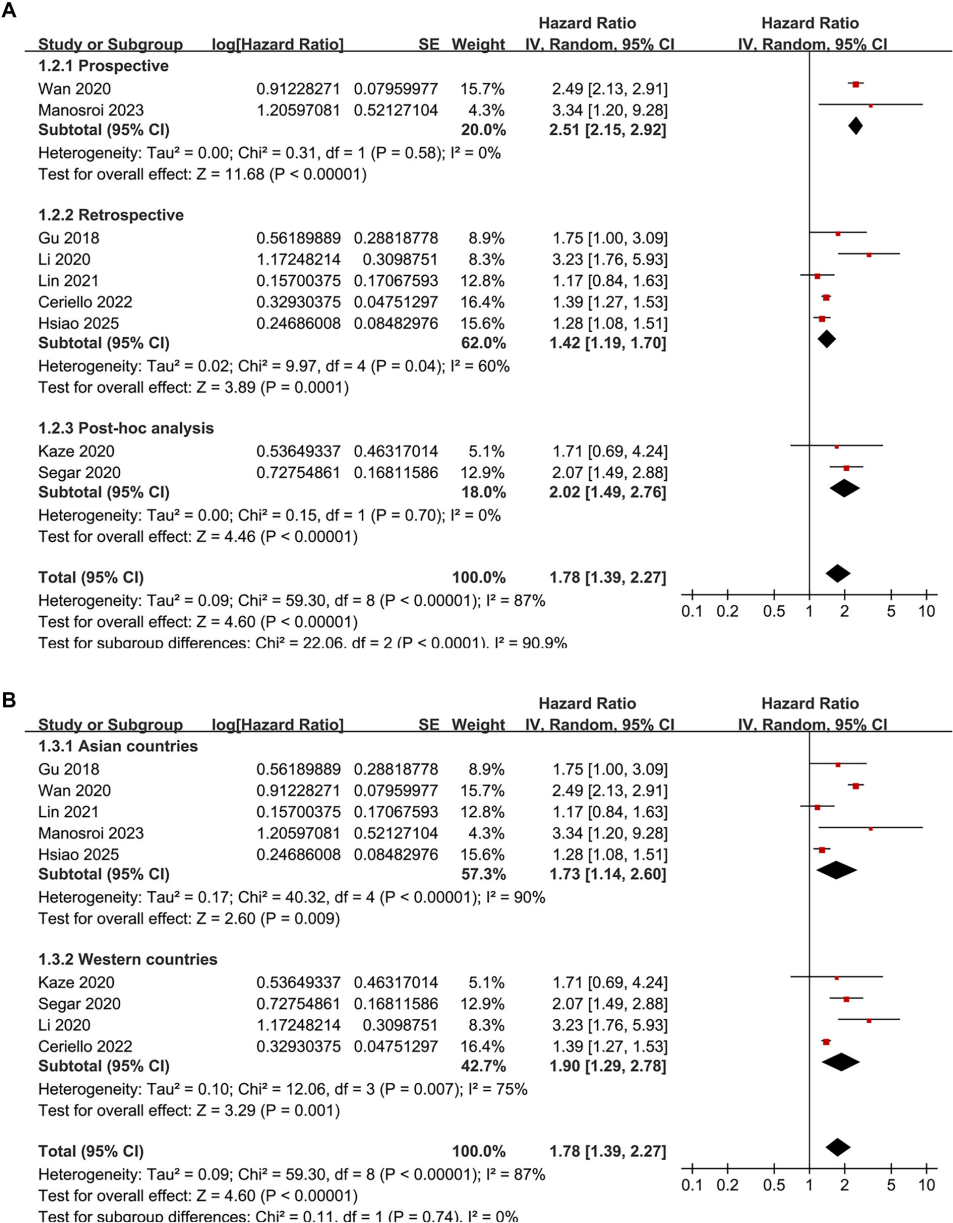
**Forest plots illustrating subgroup analyses of the association between HbA1c variability and the risk of HF.** (A) Subgroup analysis by study design; (B) Subgroup analysis by study country. Abbreviations: HbA1c: Glycated hemoglobin; HF: Heart failure.

**Figure 4.**
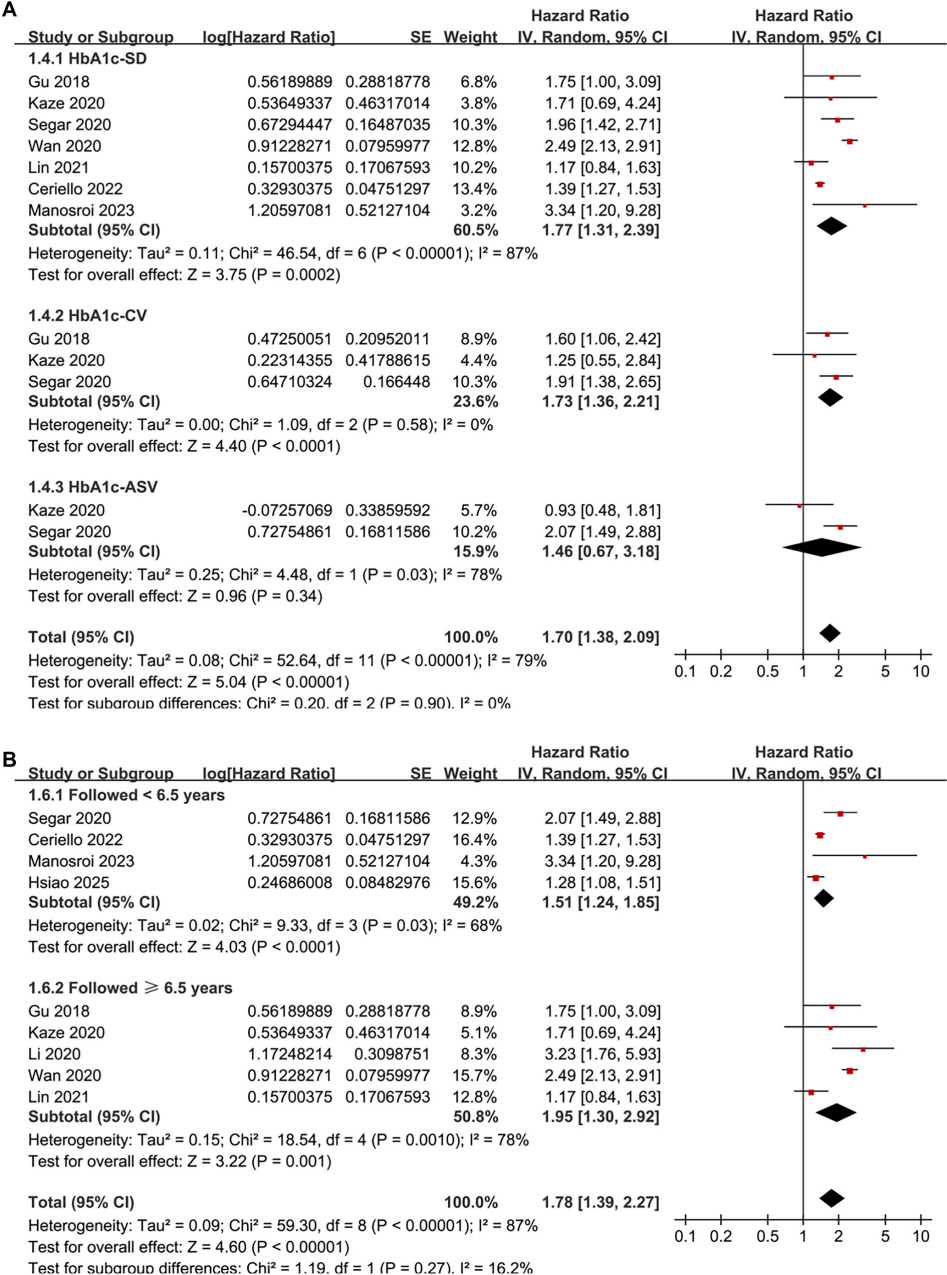
**Forest plots of subgroup analyses for the association between HbA1c variability and the risk of HF.** (A) Subgroup analysis by HbA1c-variability metric; (B) Subgroup analysis by follow-up duration. Panel A provides an exploratory, descriptive comparison of metric types; each study contributes one estimate within a given metric subgroup, whereas the primary meta-analysis (Figure 2) includes one independent estimate per study. Abbreviations: HbA1c: Glycated hemoglobin; HF: Heart failure.

Pooled results from nine studies [23–31] indicate that a high variability in HbA1c levels is associated with an increased risk of HF during follow-up (HR: 1.78, 95% CI: 1.39–2.27, *P* < 0.001; *I*^2^ ═ 87%; Figure 2). The between-study variance was τ^2^ ═ 0.09, with a 95% PI ranging from 1.01 to 3.14, suggesting that future studies are likely to report a positive association. Sensitivity analysis employing a REML random-effects model with Hartung–Knapp adjustment produced a similar effect estimate (HR: 1.78, 95% CI: 1.35–2.35, *P*< 0.001; *I*^2^ ═ 86%), **affirming** the robustness of the findings. Analyses excluding one study at a time revealed no significant impact on the results (HR: 1.57–1.90, *P* all < 0.05). Further sensitivity analyses focused solely on patients with T2D [23–29, 31] yielded consistent results (HR: 1.73, 95% CI: 1.35–2.22, *P* < 0.001; *I*^2^ ═ 88%). Similar results emerged from analyses restricted to high-quality studies (NOS ≥ 8) [23–28, 31] (HR: 1.82, 95% CI: 1.32–2.50, *P* < 0.001; *I*^2^ ═ 86%) and studies that adjusted for mean HbA1c [23, 24, 26–31] (HR: 1.68, 95% CI: 1.31–2.16, *P*< 0.001; *I*^2^ ═ 87%). Notably, subgroup analyses indicated a stronger association between high HbA1c variability and HF risk in prospective studies compared to retrospective and post-hoc studies (HR: 2.51 vs 1.42 and 2.02, *P* for subgroup difference < 0.001; Figure 3A). Similar findings were observed in studies from Asian and Western countries *(P* for subgroup difference = 0.74; Figure 3B), as well as in studies measuring HbA1c variability using SD, CV, and ASV (*P* for subgroup difference = 0.74; Figure 4A), and in studies with follow-up durations of less than 6.5 years and 6.5 years *or* more (*P* for subgroup difference = 0.27; Figure 4B). The results of the univariate meta-regression analysis are presented in Table 3. None of the predefined characteristics, including mean ages of the populations, proportions of men, mean follow-up durations, or NOS scores, significantly influenced the association between HbA1c variability and HF risk (*P* all > 0.05).

**Table 3:** Results of univariate meta-regression analysis

**Variables**	**HR for the association between HbA1c variability and the risk of heart failure**			
	**Coefficient**	**95% CI**	***P* values**	**Adjusted R^2^**
Mean age (years)	0.056	−0.079 to 0.190	0.36	0%
Men (%)	−0.0026	−0.0532 to 0.0479	0.91	0%
Follow-up duration (years)	−0.043	−0.195 to 0.109	0.53	0%
NOS	0.17	−0.34 to 0.68	0.46	0%

Abbreviations: HR: Hazard ratio; HbA1c: Glycated hemoglobin; CI: Confidence interval; NOS: Newcastle–Ottawa Scale.

### Publication bias

**Figure 5.**
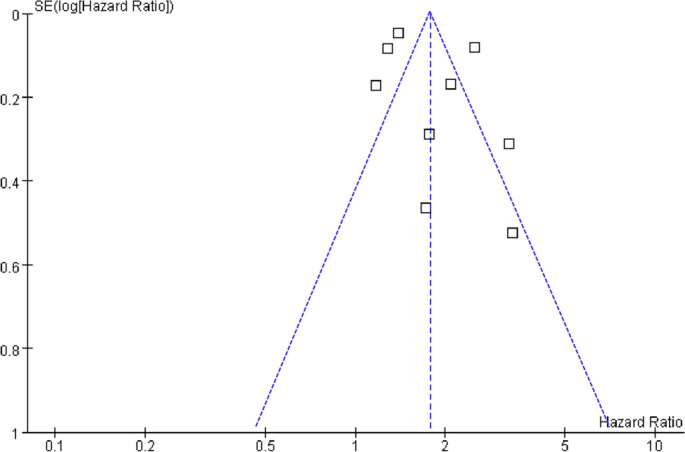
**Funnel plot for assessing potential publication bias in meta-analyses of the associations between HbA1c variability and HF risk**. Egger’s test revealed no evidence of small-study effects; however, given the limited sample of only nine studies, the results should be interpreted with caution. Abbreviations: HbA1c: Glycated hemoglobin; HF: Heart failure.

Figure 5 presents funnel plots assessing the publication bias associated with the meta-analysis on the relationship between HbA1c variability and HF risk. Visual inspection of the funnel plots suggests symmetry, indicating a low risk of publication bias. This conclusion is further supported by Egger’s regression analysis, which did not reveal significant publication bias (*P* ═ 0.35). Due to the limited number of studies (*k* ═ 9), Egger’s test has reduced power, and the lack of statistical significance should be interpreted with caution. The trim-and-fill procedure did not impute any additional studies, and the pooled HR remained unchanged, indicating no evidence of small-study effects.

## Discussion

This meta-analysis demonstrates that increased visit-to-visit variability in HbA1c levels is significantly associated with a heightened risk of developing HF, independent of mean HbA1c levels and other conventional risk factors. The consistency of findings across various sensitivity analyses—restricted to patients with T2D, high-quality studies, and those adjusting for mean HbA1c—suggests that long-term glycemic instability may hold prognostic significance beyond average glycemic exposure. The strength of this association is particularly pronounced in prospective cohorts compared to retrospective or post-hoc analyses, underscoring the robustness of data assessed over time. Collectively, these findings support the hypothesis that HbA1c variability represents an additional dimension of glycemic burden contributing to cardiovascular risk and highlights its potential role as a novel biomarker for identifying individuals at increased risk of HF.

While a recent meta-analysis has examined HbA1c variability in relation to HF risk, it did so within the context of a broader synthesis of multiple glycemic risk factors and cardiovascular outcomes [15]. In contrast, our review provides a focused and updated estimate specifically for incident HF, incorporates additional recent cohorts, and employs comprehensive subgroup and REML–Hartung–Knapp sensitivity analyses. Clinically, HbA1c variability may serve as a complementary risk marker alongside mean HbA1c, diabetes duration, and renal function, aiding in the identification of T2D patients who may require closer monitoring, earlier initiation of HF-preventive therapies, or more stable glucose-lowering strategies. Several biological mechanisms may elucidate the connection between long-term HbA1c variability and increased HF risk. Repeated fluctuations in glucose levels have been shown to induce greater oxidative stress and endothelial dysfunction compared to sustained hyperglycemia, leading to impaired nitric oxide bioavailability and microvascular inflammation [32–34]. These fluctuations can promote maladaptive cardiac remodeling through the accumulation of advanced glycation end-products (AGEs), mitochondrial dysfunction, and activation of pro-fibrotic pathways [35–37]. In diabetic cardiomyopathy, intermittent hyperglycemia triggers sympathetic overactivity and metabolic inflexibility, resulting in impaired myocardial energy utilization and left ventricular diastolic dysfunction—key precursors of HF [38]. Furthermore, HbA1c variability may reflect fluctuations in treatment adherence or medication responsiveness, potentially exacerbating cardiovascular instability [39]. Collectively, these mechanisms suggest that maintaining stable long-term glycemic control may be as critical as reducing mean HbA1c for the prevention of HF.

The results of subgroup and sensitivity analyses provide additional insights into the robustness and potential heterogeneity of this association. The stronger relationship observed in prospective cohorts likely reflects the more rigorous data collection and outcome validation inherent to such designs, reducing the risk of measurement bias. Studies that adjusted for mean HbA1c retained a significant association, supporting the hypothesis that glycemic fluctuation exerts harmful cardiovascular effects beyond chronic hyperglycemia itself [40]. Similarly, consistent findings across high-quality studies and those with extended follow-up durations reinforce the temporal plausibility of the association. The absence of significant modifiers in the meta-regression analysis—including age, sex, study quality, and follow-up duration—suggests that the detrimental impact of HbA1c variability may be broadly consistent across populations, although subtle interactions may exist that cannot be detected without individual-level data.

From a clinical perspective, these findings underscore the potential value of incorporating measures of HbA1c variability into long-term risk assessment frameworks for patients with T2D. Current diabetes management guidelines primarily emphasize mean HbA1c targets [41]. However, these results suggest that minimizing long-term fluctuations in glycemia may offer additional cardiovascular benefits [41]. Regular monitoring of HbA1c variability could facilitate the identification of high-risk individuals who may benefit from more consistent glycemic control strategies, enhanced medication adherence, or early interventions for cardiovascular risk reduction. Clinicians should be aware that treatment regimens with a higher propensity for glycemic oscillation—such as those involving short-acting insulin secretagogues or intermittent insulin dosing—may increase the risk of adverse cardiac outcomes if glycemic variability is not effectively managed [42, 43].

This meta-analysis possesses several strengths that enhance the credibility of its findings. First, it serves as an updated summary of the evidence, encompassing nine longitudinal cohorts with over 340,000 participants and more than 10,000 incident HF events. Second, all included studies employed longitudinal designs with temporally defined exposure and outcome periods, ensuring that HbA1c variability preceded the onset of HF. Third, the analyses utilized multivariable-adjusted risk estimates, thereby minimizing confounding by established cardiovascular risk factors. Additionally, multiple sensitivity and subgroup analyses confirmed the stability of the results across diverse study settings, designs, and analytic approaches.

Nonetheless, several limitations must be acknowledged. The majority of the included studies focused on patients with T2D, leaving the association between HbA1c variability and HF risk in individuals without diabetes or with type 1 diabetes uncertain. While two post-hoc analyses were derived from randomized clinical trials [24, 26], most studies were observational, and the possibility of residual and time-varying confounding cannot be excluded. Significant heterogeneity was observed among studies, likely due to variations in definitions and metrics of HbA1c variability (e.g., SD, CV, ARV, and VIM), population characteristics, comorbidity profiles, and concurrent medication use. Because individual participant data were unavailable, the influence of certain confounding variables—particularly antidiabetic treatment type, treatment adherence, and changes in therapy over time—could not be fully examined. Furthermore, as the included studies were based on observational data, causality cannot be established, and reverse causation remains a possibility despite efforts to exclude participants with preexisting HF. Additionally, definitions of HF varied across studies; only a subset employed fully adjudicated clinical endpoints, while others relied on validated registry or administrative codes. Because adjudicated outcomes were available in only a minority of cohorts, we could not conduct a sensitivity analysis restricted to adjudicated HF, which should be considered when interpreting the findings. Some studies used registry or administrative data to define HF outcomes, potentially introducing misclassification bias, although sensitivity analyses indicated overall robustness of the findings. Finally, a dose–response meta-analysis could not be performed due to most studies reporting HbA1c variability in categorical form (e.g., quartiles or tertiles) without providing continuous effect estimates per SD or unit increase, limiting our ability to harmonize exposure thresholds. Consequently, our clinical interpretation relies on relative hazards and the PI, emphasizing the need for future studies to report standardized absolute risk measures to facilitate clinical translation.

Given these limitations, the results should be interpreted with caution. Future research should aim to validate these findings in non-diabetic and type 1 diabetic populations and explore the pathophysiological mechanisms underlying the observed relationship using longitudinal studies with standardized definitions of HbA1c variability. Individual participant data meta-analyses would enable more refined analyses adjusting for medication use, comorbidities, and time-dependent changes in glycemia. Randomized trials evaluating interventions designed to minimize long-term glycemic fluctuations may ultimately clarify whether reducing HbA1c variability can lead to improved cardiovascular outcomes, including the prevention of HF.

## Conclusion

In conclusion, this meta-analysis suggests that greater visit-to-visit HbA1c variability may be independently associated with an increased risk of incident HF among adults, particularly those with T2D. These findings emphasize the importance of maintaining stable long-term glycemic control, in addition to achieving optimal mean HbA1c levels, as part of comprehensive cardiovascular risk management. Given that all included data are derived from observational studies, the overall certainty of evidence for this association should be regarded as low to moderate, and the findings should be interpreted accordingly. Further research is warranted to determine whether strategies aimed at reducing glycemic variability can effectively lower HF risk and improve long-term outcomes in diabetic populations.

## Supplemental data

**Supplemental file 1.** **Detailed search strategy for each database**

**PubMed**

**1. Population/exposure (HbA1c/glycemic terms)** (“Glycated Hemoglobin A”[Mesh] OR “Hemoglobin A, Glycosylated”[tiab] OR “Hemoglobin A1c”[tiab] OR HbA1c[tiab] OR A1c[tiab] OR “glycated hemoglobin”[tiab] OR “glycosylated hemoglobin”[tiab] OR glucose[tiab] OR glycemic[tiab])

**2. Variability (visit-to-visit/dispersion metrics)** (variab*[tiab] OR fluctuat*[tiab] OR “visit-to-visit”[tiab] OR “visit to visit”[tiab] OR intervisit[tiab] OR intraindividual[tiab] OR “intra-individual”[tiab] OR “within-person”[tiab] OR “within person”[tiab] OR “coefficient of variation”[tiab] OR CV[tiab] OR “standard deviation”[tiab] OR SD[tiab] OR “average real variability”[tiab] OR ARV[tiab] OR “adjacent standard deviation”[tiab] OR ASV[tiab] OR “variability independent of the mean”[tiab] OR VIM[tiab])

**3. Outcome (Heart failure)** (“Heart Failure”[Mesh] OR “Ventricular Dysfunction, Left”[Mesh] OR “Heart Failure”[tiab] OR “cardiac failure”[tiab] OR “cardiac dysfunction”[tiab] OR “ventricular dysfunction”[tiab] OR “left ventricular dysfunction”[tiab])

**4. Study design/incidence/risk** (“Incidence”[Mesh] OR incidence[tiab] OR risk[tiab] OR hazard*[tiab] OR cohort*[tiab] OR longitudinal[tiab] OR prospective[tiab] OR retrospective[tiab] OR “follow-up”[tiab] OR followed[tiab])

**5. Combine and date limit** 1 AND 2 AND 3 AND 4 AND (“0001/01/01”[Date - Publication]: “2025/08/30”[Date - Publication])

**Embase**

**1. HbA1c/glycemic** ‘glycated hemoglobin a’/exp OR ‘hemoglobin a1c’/exp OR (hba1c OR ‘hemoglobin a1c’ OR ‘glycated hemoglobin’ OR ‘glycosylated hemoglobin’ OR glucose OR glycemic):ti,ab,kw

**2. Variability/metrics** (variab* OR fluctuat* OR ‘visit-to-visit’ OR (visit NEAR/2 visit) OR intervisit OR ‘intra-individual’ OR intraindividual OR ‘within-person’ OR ‘within person’ OR ‘coefficient of variation’ OR CV OR ‘standard deviation’ OR SD OR ‘average real variability’ OR ARV OR ‘adjacent standard deviation’ OR ASV OR ‘variability independent of the mean’ OR VIM):ti,ab,kw

**3. Heart failure** ‘heart failure’/exp OR ‘left ventricular dysfunction’/exp OR (‘cardiac’ NEAR/2 (failure OR dysfunction)):ti,ab,kw OR ‘ventricular dysfunction’/exp

**4. Study design/incidence/risk** ‘incidence’/exp OR ‘risk’/exp OR ‘cohort analysis’/exp OR ‘longitudinal study’/exp OR ‘prospective study’/exp OR ‘retrospective study’/exp OR ‘follow up’/exp OR (cohort* OR longitudinal OR prospective OR retrospective OR ‘follow-up’ OR followed):ti,ab,kw

**5. Combine and years** 1 AND 2 AND 3 AND 4, from inception to **2025-08-30**

**Web of Science**

TS=((“hemoglobin a1c” OR HbA1c OR A1c OR “glycated hemoglobin” OR “glycosylated hemoglobin” OR glucose OR glycemic) AND (variab* OR fluctuat* OR “visit-to-visit” OR (visit NEAR/2 visit) OR intervisit OR “intra-individual” OR intraindividual OR “within-person” OR “within person” OR “coefficient of variation” OR CV OR “standard deviation” OR SD OR “average real variability” OR ARV OR “adjacent standard deviation” OR ASV OR “variability independent of the mean” OR VIM) AND (“heart failure” OR (“cardiac” NEAR/2 (failure OR dysfunction)) OR “ventricular dysfunction” OR “left ventricular dysfunction”) AND (incidence OR risk OR hazard* OR cohort* OR longitudinal OR prospective OR retrospective OR “follow-up” OR followed))

(Indexes: SCI-EXPANDED, SSCI, A&HCI, CPCI-S, CPCI-SSH, ESCI; Timespan: 1900–2025; Language: All)

**Figure S1.**
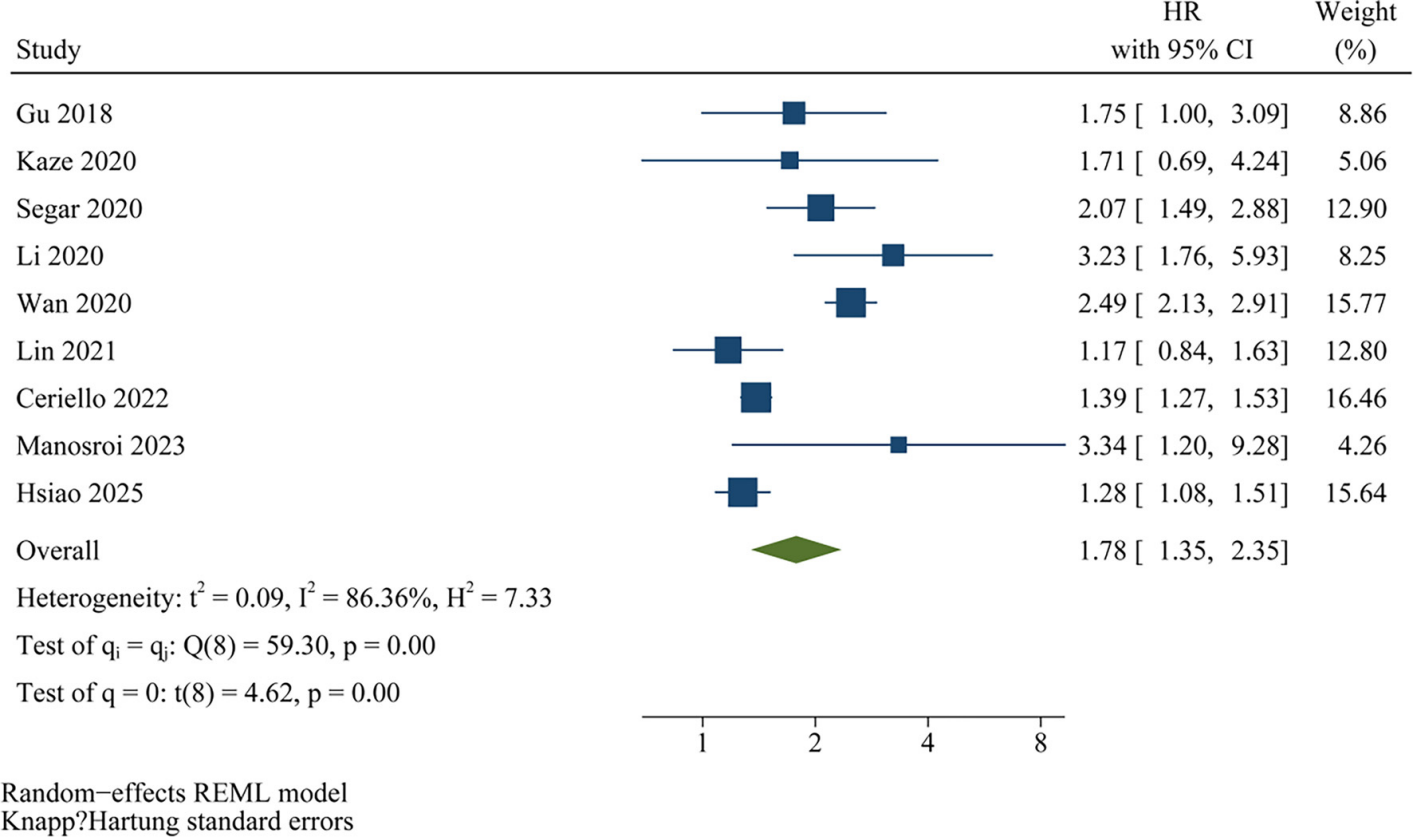
**Sensitivity analysis of the association between high vs. low visit-to-visit HbA1c variability and incident HF using a REML random-effects model with Hartung–Knapp inference.** Pooled estimates are shown as HRs with 95% CIs, demonstrating results consistent with the primary analysis (HR 1.78, 95% CI 1.35–2.35; *I*^2^ ═ 86%). Abbreviations: HbA1c: Glycated hemoglobin; HF: Heart failure; HR: Hazard ratio; CI: Confidence interval; REML: Restricted maximum likelihood.

## Data Availability

All data generated or analyzed during this study are included in this published article.
